# The importance of hippocampal dynamic connectivity in explaining memory function in multiple sclerosis

**DOI:** 10.1002/brb3.954

**Published:** 2018-03-30

**Authors:** Quinten van Geest, Hanneke E. Hulst, Kim A. Meijer, Lieke Hoyng, Jeroen J.G. Geurts, Linda Douw

**Affiliations:** ^1^ Department of Anatomy & Neurosciences VU University Medical Center Amsterdam Neuroscience VUmc MS Center Amsterdam Amsterdam The Netherlands; ^2^ Department of Radiology Athinoula A. Martinos Center for Biomedical Imaging Massachusetts General Hospital Charlestown MA USA

**Keywords:** cognition, dynamic functional connectivity, functional magnetic resonance imaging, hippocampus, memory, multiple sclerosis

## Abstract

**Introduction:**

Brain dynamics (i.e., variable strength of communication between areas), even at the scale of seconds, are thought to underlie complex human behavior, such as learning and memory. In multiple sclerosis (MS), memory problems occur often and have so far only been related to “stationary” brain measures (e.g., atrophy, lesions, activation and stationary (s) functional connectivity (FC) over an entire functional scanning session). However, dynamics in FC (dFC) between the hippocampus and the (neo)cortex may be another important neurobiological substrate of memory impairment in MS that has not yet been explored. Therefore, we investigated hippocampal dFC during a functional (f) magnetic resonance imaging (MRI) episodic memory task and its relationship with verbal and visuospatial memory performance outside the MR scanner.

**Methods:**

Thirty‐eight MS patients and 29 healthy controls underwent neuropsychological tests to assess memory function. Imaging (1.5T) was obtained during performance of a memory task. We assessed hippocampal volume, functional activation, and sFC (i.e., FC of the hippocampus with the rest of the brain averaged over the entire scan, using an atlas‐based approach). Dynamic FC of the hippocampus was calculated using a sliding window approach.

**Results:**

No group differences were found in hippocampal activation, sFC, and dFC. However, stepwise forward regression analyses in patients revealed that lower dFC of the left hippocampus (standardized β = −0.30; *p *=* *.021) could explain an additional 7% of variance (53% in total) in verbal memory, in addition to female sex and larger left hippocampal volume. For visuospatial memory, lower dFC of the right hippocampus (standardized β = −0.38; *p *=* *.013) could explain an additional 13% of variance (24% in total) in addition to higher sFC of the right hippocampus.

**Conclusion:**

Low hippocampal dFC is an important indicator for maintained memory performance in MS, in addition to other hippocampal imaging measures. Hence, brain dynamics may offer new insights into the neurobiological mechanisms underlying memory (dys)function.

## 1 INTRODUCTION

Problems with cognitive functioning, such as learning and memory problems, occur frequently in multiple sclerosis (MS) (Chiaravalloti & DeLuca, 2008; Rao, Leo, Bernardin, & Unverzagt, 1991) and have a tremendous impact on patients’ quality of life (Mitchell, Benito‐Leon, Gonzalez, & Rivera‐Navarro, 2005). Neuroimaging has explored “traditional” brain correlates of these memory problems, such as gray matter (GM) volume, brain activation, and stationary (s) functional connectivity (FC) of particularly the hippocampus during functional (f) magnetic resonance imaging (MRI) (Benedict, Ramasamy, Munschauer, Weinstock‐Guttman, & Zivadinov, 2009; Hulst et al., 2015; Roosendaal et al., 2008, 2010; Steenwijk et al., 2016). Hippocampal activation during task fMRI seems most specific, as it also shows lateralization in terms of content specificity: The left hippocampus is mainly involved in verbal learning and memory, while the right hippocampus predominantly associates with visuospatial learning and memory (Avila et al., 2006; Igloi, Doeller, Berthoz, Rondi‐Reig, & Burgess, 2010). Unfortunately, learning and memory function is still poorly understood mechanistically, and its deterioration in patients with MS remains challenging to track or predict with abovementioned neuroimaging parameters. Dynamic coupling between functionally connected brain regions, that is, variable strength of communication between areas (from now on termed *brain dynamics*), is a recently described and possibly seminal substrate of complex human cognition in health and disease (Hutchison et al., 2013). Hence, dynamic measures of FC might provide a new layer of temporal information on processes possibly underlying learning and memory (dys)function in MS.

Recent studies indicate that the dynamics of FC (dFC) are indeed related to cognitive function in healthy subjects and certain patient populations (Cole et al., 2013; Douw, Wakeman, Tanaka, Liu, & Stufflebeam, 2016; Douw et al., 2015; Hellyer, Jachs, Clopath, & Leech, 2016; Nguyen et al., 2016), and that dFC may supersede traditional neuroimaging measures in explaining cognitive variance (Douw et al., 2015, 2016; Jia, Hu, & Deshpande, 2014; Nguyen et al., 2016). This body of literature is heterogeneous, but suggests that the relationship between dFC and cognition depends on the combination of the brain state in which it is measured (at rest or during a task) and the cognitive domain investigated. With respect to brain state in healthy subjects, the brain seems to display different levels of dFC and sFC in a focused (during a task) versus unfocused state (at rest) (Hellyer et al., 2016). For example, during performance of a demanding reaction time task, a decrease in global brain dynamics and an increase in sFC as compared to the resting state (RS) can be observed (Hellyer et al., 2016). This could imply that strong and stable connectivity (high sFC, low dFC) is necessary for task execution. However, during performance of an executive functioning task, an opposite relationship has been found, namely a positive relationship between dFC of the frontoparietal network (important for cognitive control) and in‐scanner cognitive performance (Cole et al., 2013) and executive functioning outside the scanner (Douw et al., 2016). Additionally, during simple motor learning, dynamics of certain brain regions seem to be a predictor for learning effects in a future session in healthy subjects (Bassett et al., 2011). These results show that the link between brain dynamics and cognition relies on the brain state (task vs rest) and cognitive domain under investigation.

Also in neurological disorders, brain dynamics seem to be of relevance for behavior. For example, in temporal lobe epilepsy, decreased RS dFC of the posterior cingulate cortex has been linked to poorer memory performance (Douw et al., 2015), whereas in bipolar disorder lower dFC between the posterior cingulate cortex and medial prefrontal cortex was linked to slower processing speed and reduced cognitive set‐shifting (Nguyen et al., 2016). Additionally, altered dFC of frontal and temporal regions can identify subjects with mild cognitive impairment (Chen et al., 2017), whereas in minimally disabled patients with MS, dFC of parietal and prefrontal regions is altered compared to healthy subjects, but not linked to physical disability (Leonardi et al., 2013). Although previous studies offer ample evidence for the importance of brain dynamics for our understanding of human cognition in health and disease, they primarily focus on the RS and/or executive functioning. The association between hippocampal dFC during active recruitment of the memory system and learning and memory function measured outside the scanner has not been investigated yet, but may offer fundamental insight into how dynamics of the functional hippocampal network underlies learning and memory function, particularly in patients with MS suffering from deficits in this cognitive domain.

Therefore, we investigated verbal and visuospatial learning and memory in a group of MS patients and healthy subjects. We hypothesize that: (1) dFC of the hippocampus during a visuospatial memory task is different in patients with MS compared to healthy controls (HCs), (2) dFC explains additional variance in verbal and visuospatial learning and memory performance outside the scanner on top of traditional brain measures (i.e., atrophy, hippocampal activation, and sFC), and; (3) a lateralization effect is present for dFC and verbal and visuospatial learning and memory function, such that verbal and visuospatial learning and memory performance can be explained by dFC of the left and right hippocampus, respectively.

## 2 MATERIALS AND METHODS

### 2.1 Subjects

All subjects were part of a previously published fMRI study (Hulst et al., 2012) and met the following inclusion criteria: (1) no contra‐indications for MRI; (2) aged between 18 and 65 years, and; (3) no psychiatric or neurological diseases (for patients: other than MS). Additionally, subjects with many large frame‐to‐frame head movements (≥5 movements of >0.5 mm) during the fMRI task were excluded to minimalize motion effects. Only patients with relapsing‐remitting and secondary progressive MS were included. All patients were diagnosed according to the revised McDonald criteria (Polman et al., 2011), and relapse‐free and without steroid treatment for at least 6 weeks. The study protocol was approved by the institutional ethical review board and conducted in accordance with the ethical standards laid down in the Declaration of Helsinki. Written informed consent was obtained from each participant.

### 2.2 Learning and memory performance and self‐report questionnaires

All participants underwent neuropsychological testing, including tests for information processing speed, working memory, verbal fluency, and verbal and visuospatial learning and memory (see a previous publication for a detailed description of all neuropsychological tests) (Hulst et al., 2012). Verbal learning and memory was assessed with the Verbale Leer‐ en Geheugen Taak (Mulder, Dekker, & Dekker, 1996), which is the Dutch equivalent of the California Verbal Learning Test. In this task, a grocery shopping list with 16 items is verbally presented to the participant five times. After each presentation, the subject has to recall as many items as possible, and the total number of recalled items is the score. Visuospatial learning and memory performance was measured by the Location Learning Test (Bucks & Willison, 1997). In this test, a five‐by‐five grid on which ten different items are displayed, is presented for 15 s. During those 15 s, the subject has to learn the location of each item. After 15 s, an empty grid is presented and cards representing each item are provided to the subject one after the other, which he or she has to put in the correct location. This learning phase is repeated five times, and the score reflects the total number of displacements over all five trials (the higher the score, the poorer the performance). The raw test scores were converted into Z‐scores relative to HCs. For the sake of clarity, the Z‐score for the Location Learning Test was inverted.

Physical disability of patients was assessed using the telephone version of the Expanded Disability Status Scale (Lechner‐Scott et al., 2003). Levels of anxiety and depression were measured in all subjects by the Hospital Anxiety and Depression Scale (HADS‐A/D) (Zigmond & Snaith, 1983), and fatigue was measured with the Checklist of Individual Strength (Vercoulen et al., 1994).

### 2.3 MRI acquisition

All subjects underwent MR scanning at 1.5T (Siemens Sonata, Erlangen, Germany) using an eight‐channel phased array head coil. High‐resolution three‐dimensional T1‐weighted (3DT1) images were obtained with magnetization prepared rapid acquisition gradient‐echo (repetition time (TR): 2,700 ms; echo time (TE): 5 ms; inversion time (TI): 950 ms; 176 sagittal slices with 1.3 mm thickness; field of view (FOV): 248 × 330 mm^2^; 1.3 × 1.3 mm^2^ in plane resolution). A turbo spin‐echo proton density/T2‐weighted scan (TR: 3,130 ms; TE: 24/85 ms; 46 axial slices; FOV: 192 × 256 mm^2^ with 3.0 mm slice thickness; 1.0 × 1.0 mm^2^ in plane resolution) was acquired for white matter lesion quantification. Three‐dimensional double inversion recovery (DIR) imaging (TR: 2,350 ms; TE: 355 ms; TI: 350 ms; 120 sagittal slices; FOV: 192 × 256 mm^2^ with 1.2 mm slice thickness; 1.2 × 1.2 mm^2^ in plane resolution) was acquired in patients for hippocampal lesion detection. Task‐related fMRI consisted of 208 volumes (partial brain coverage) of echo‐planar images (TR: 2,220 ms; TE: 60 ms; 28 axial slices with 3 mm slice thickness; FOV: 211 × 211 mm^2^; 3.3 × 3.3 mm^2^ in plane resolution). In order to optimize registration of the task‐related fMRI data, one whole‐brain volume of the subject's RS scan (200 volumes of echo‐planar images; TR: 2,850 ms; TE: 60 ms; 36 axial slices with 3.3 mm slice thickness; FOV: 211 × 211 mm^2^; 3.3 × 3.3 mm^2^ in plane resolution) was used.

### 2.4 Structural MRI processing

Processing of MRI data was performed using FSL5 (FMRIB's Software Library, http://www.fmrib.ox.ac.uk/fsl). GM and white matter (WM) segmentation was performed using SienaX (Smith et al., 2002). Subcortical brain structures were segmented using FIRST (Patenaude, Smith, Kennedy, & Jenkinson, 2011). Brain volumes were normalized for head size, resulting in normalized GM volume (NGMV), normalized WM volume (NWMV), and normalized hippocampal volume (NHV). For descriptive purposes and not used in further analyses, an experienced rater (H.E. Hulst) counted the number of hippocampal lesions for each patient (one patient did not have a DIR sequence). On the proton density/T2‐weighted images, WM lesions in patients were manually outlined using a local threshold technique. No lesion filling was performed.

### 2.5 Functional MRI paradigm

The fMRI paradigm used in this study has been described and analyzed previously in terms of brain activation (Hulst et al., 2012), but not in terms of sFC and dFC. For this study, we used the encoding phase of the episodic memory paradigm and not the retrieval phase, because the hippocampus has shown to be active especially during encoding (Van Der Werf et al., 2009). In short, the subject was shown 50 different novel landscape images for 5 s. During stimulus presentation, the subject had to indicate whether the landscape was tropical or nontropical by pressing a button (see Figure S1). This assignment ensured that the attention was directed toward the image and its details, which is thought to facilitate correct encoding and has been shown to enhance hippocampal activation (Daselaar, Veltman, Rombouts, Raaijmakers, & Jonker, 2003). The order of images was prerandomized and alternated with 20 control images, in which the subject had to indicate whether the arrow shown on a familiar landscape image pointed to the left or right. In the retrieval phase (approximately 30 min after the encoding phase; fMRI data not used in this study), old and novel landscape images were presented in a prerandomized order. The participant had to indicate whether or not he or she recognized the presented landscape image, enabling us to calculate the number of correctly encoded landscape images.

### 2.6 Functional MRI measures

#### 2.6.1 Hippocampal activation

Detailed information concerning the measure of hippocampal activation has been described previously (Hulst et al., 2012). In short, brain activation during correctly encoded items was contrasted to brain activation during the control arrow condition using FEAT (Beckmann, Jenkinson, & Smith, 2003). Next, for each subject, the average signal, expressed in a Z‐value, for the right and left hippocampus was obtained.

#### 2.6.2 fMRI preprocessing and atlas development

Preprocessing of the fMRI data was performed with Melodic, and consisted of: (1) discarding the first five volumes; (2) motion correction; (3) spatial smoothing (6 mm full width‐at‐half‐maximum Gaussian kernel), and; (4) high pass filtering (1.0 s cutoff) (Beckmann & Smith, 2004). The fMRI data were registered to standard space, using a three‐step registration. The low‐resolution task‐related fMRI scan (partial brain coverage; the part superior of the cingulate cortex was usually absent) was first registered to a whole‐brain RS fMRI volume, which was subsequently registered to the 3DT1 scan. Next, the 3DT1 scan was registered to MNI152 standard space. For each subject, the inverse of the aforementioned registration matrix was applied to the Automated Anatomical Labeling atlas (Tzourio‐Mazoyer et al., 2002), in order to create individual brain atlases. Next, we masked this individual atlas for GM, and subsequently added the subcortical structures. This results in an atlas consisting of 92 cortical and subcortical brain regions in 3DT1 space which was then registered to the subject's fMRI space. Next, for each subject, a fMRI mask was constructed that excluded areas known to be prone to artifacts (e.g., orbitofrontal cortex), by excluding voxels with a signal intensity in the lowest quartile of the robust intensity range. This mask was multiplied with the atlas, and for each remaining atlas region, we obtained the mean time series. Additionally, average head motion during the fMRI task was obtained from Melodic for each subject.

#### 2.6.3 Hippocampal sFC

The time series were imported into Matlab R2012a (Natick, Massachusetts, USA) and processed to obtain sFC and dFC measures of the entire brain and the left and right hippocampus. Missing atlas regions due to partial brain coverage and/or masking for fMRI artifacts were coded as missing. To obtain sFC, Pearson correlation coefficients were used to correlate activity between all regions over the entire time series (absolute values). This resulted in one sFC matrix of 92 by 92 for each subject. From this matrix, sFC of the entire brain as well as for the left and right hippocampus with the rest of the brain was obtained.

#### 2.6.4 Hippocampal dFC

**Figure 1:**
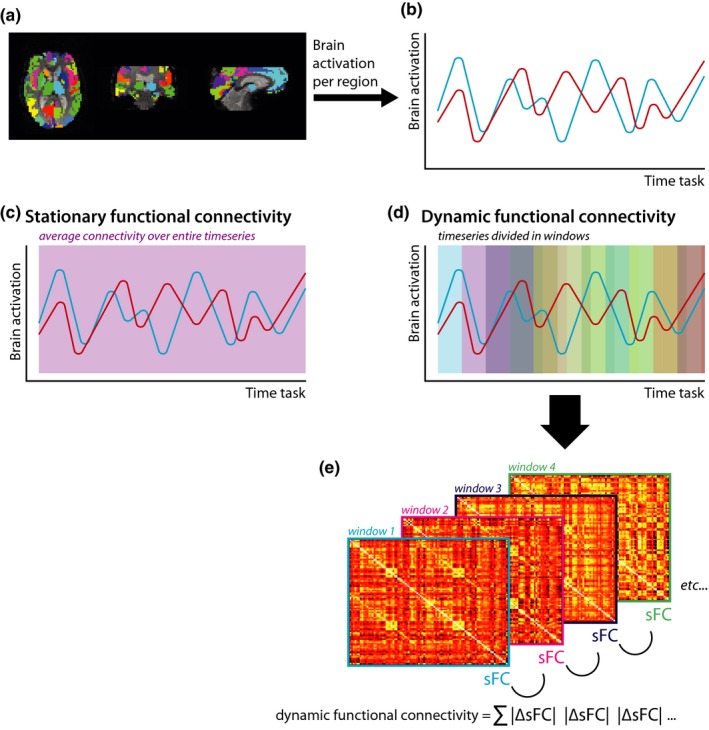
Schematic overview of stationary and dynamic functional connectivity analysis pipeline. For each cortical and subcortical brain region (a), the mean time series were obtained (b). Stationary functional connectivity was calculated over the entire time series (c), while for dFC the time series were divided into sliding windows (d). For each sliding window, the stationary functional connectivity was calculated, and subsequently, the absolute difference between each consecutive window was calculated and summed as a measure for dFC (e). sFC, stationary functional connectivity; dFC, dynamic functional connectivity

Dynamic FC was measured using a sliding window approach (see Figure 1). Based on previous studies (Douw et al., 2016; Leonardi, Shirer, Greicius, & Van De Ville, 2014; Leonardi & Van De Ville, 2015), the fMRI time series were divided into 35 sliding windows with a length and shift of 59.9 and 11.1 s, respectively. For each window, sFC was calculated. Next, the absolute difference in sFC was calculated between each consecutive window. Subsequently, these absolute differences were summed, resulting in one matrix of 92 by 92 elements that contained the summed differences in sFC for each node pair over all sliding windows. High values indicate large variations in connectivity strength over time during the task (i.e., high dFC). In contrast, low values imply there is little variation in connectivity strength and thus low dFC.

Hippocampal sFC and dFC were normalized by the corresponding whole‐brain averages, in order to be more specific to detect regional changes in these measures, instead of only picking up global between‐subject differences in sFC and dFC.

### 2.7 Statistics

All statistical analyses were performed in SPSS 20 (Chicago, IL, USA). Normality of data was assessed with the Kolmogorov–Smirnov test and visual inspection of histograms. For normally distributed data, group differences were tested using univariate or multivariate analyses of variance ((M)ANOVA), whereas for non‐normally distributed data the Kruskal–Wallis test was used. Group differences in sFC and dFC measures were tested using a MANOVA with average head motion as a covariate, as motion can affect sFC and dFC measures.

Hippocampal dFC was correlated with hippocampal activation and sFC using Pearson correlation coefficients, in order to investigate to what extent dFC is separable from other functional measures. Furthermore, dFC of the hippocampus was correlated with several measures of disease progression (i.e., hippocampal volume, WM lesion volume, head motion, and disease duration), anxiety and depression (HADS‐A/D), and fatigue to ensure dFC was not related to any of these variables (for non‐normally distributed data, Spearman rank correlation coefficient was used). To investigate whether hippocampal dFC in patients could explain additional variance in learning and memory performance outside the scanner, stepwise regression analyses with forward selection were performed. Z‐scores of verbal and visuospatial learning and memory were predicted separately using different blocks containing relevant predictors based on previous studies to be able to quantify the added value of each measure in terms of explained variance. The included predictors were: block 1: age, sex, educational level, anxiety, depression, and fatigue (possible confounding variables for learning and memory); block 2: volume left and right hippocampus; block 3: task‐related fMRI signal of the left and right hippocampus; block 4: sFC of the left and right hippocampus; and block 5: dFC of the left and right hippocampus. *p*‐values <.05 were considered statistically significant for all these analyses.

We visually explored the importance of each dynamic hippocampal connection for verbal and visuospatial memory separately in a post hoc analysis. For each connection, the increase in effect size by adding dFC on top of stationary measures was calculated (Cohen's f^2^), and subsequently projected on a glass brain in Matlab (using BrainNet Viewer) (Xia, Wang, & He, 2013).

#### 2.7.1 Specificity analyses

In order to investigate the specificity of our findings with respect to the relevance of hippocampal dFC for learning and memory, we reran the abovementioned regression analyses and substituted dFC of the hippocampus with dFC of the thalamus (bilateral). Furthermore, we repeated the regression analyses and added bilateral thalamic dFC, NWMV, or NGMV next to hippocampal dFC (in block 5). Finally, we ran a regression analysis in which we predicted average cognitive functioning (average Z‐score of all investigated cognitive domains) using hippocampal measures.

## 3 RESULTS

### 3.1 Demographics, learning and memory performance and self‐report questionnaires

Due to excessive head motion (≥5 movements of >0.5 mm during the memory encoding task), 12 patients with MS and one HC were excluded, resulting in a final sample size of 38 patients (26 female; mean age 47.2 ± 8.0 years; average disease duration 11.2 ± 7.2 years) and 29 HCs (18 female; mean age 43.9 ± 8.4 years). Table 1 summarizes the demographic, learning and memory performance and self‐report questionnaire data. No group differences were found concerning age (*F *=* *2.66; *p *=* *.108), sex (*Χ* ^2^ = 2.66; *p *=* *.587), and educational level (*U *=* *531; *p *=* *.785), although MS patients reported higher levels of anxiety, depression, and fatigue (*p *<* *.01 for all). Verbal and visuospatial learning and memory performance were poorer in patients compared to HCs (*U *=* *363.5; *p *=* *.018, and *U *=* *289; *p *=* *.001, respectively). In total, 12 patients met the criteria for cognitive impairment (scoring at least two standard deviations below HCs on at least two cognitive domains), and the other 26 patients were defined as cognitively preserved.

**Table 1:** Demographics, self‐report questionnaires, and learning and memory performance of patients with multiple sclerosis and healthy controls

	MS patients (*n *=* *38)	HCs (*n *=* *29)	*p*
Age, years	47.19 ± 8.01	43.90 ± 8.40	.108
F/M	26/12	18/11	.587^b^
Educational level^a^	6.00 (5.00–6.00)	6.00 (5.00–6.00)	.785
RRMS/SPMS	30/8	–	–
Disease duration, years	11.24 ± 7.16	–	–
EDSS^a^	3.50 (3.50–4.50)	–	–
HADS‐A^a^	6.00 (4.00–10.00)	3.00 (2.00–6.00)	.002
HADS‐D^a^	4.00 (3.00–7.25)	1.00 (0.00–2.50)	<.001
CIS‐20^a^	77.50 (57.00–89.25)	26.00 (16.50–47.00)	<.001
Z‐score verbal learning and memory^a^	−0.50 (−1.71–0.19)	0.16 (−0.78–0.83)	.018
Z‐score visuospatial learning and memory^a^	−0.77 (−2.45–0.07)	0.30 (−0.26–0.86)	.001
Number of cognitively impaired patient	12	–	–

Displayed data are mean ± *SD* for normally distributed variables. For non‐normally distributed data, median (interquartile range) is provided.

A, anxiety; CIS‐20, Checklist of Individual Strength; D, depression; EDSS, Expanded Disability Status Scale; F, female; HADS, Hospital Anxiety and Depression Scale; M, male; RRMS, relapsing‐remitting multiple sclerosis; SPMS, secondary progressive multiple sclerosis.

a Non‐normally distributed variable.

b Chi‐square test.

### 3.2 Structural MRI

Both NGMV and NWMV were lower in patients with MS compared to HCs (*p *≤* *.001; see Table 2). Additionally, NHV (bilateral) was smaller in patients compared to HCs (*p *<* *.01). In patients, the median number of hippocampal lesions was 1.

**Table 2:** Structural MRI data from patients with multiple sclerosis and healthy controls

	MS patients (*n *=* *38)	HCs (*n *=* *29)	Test statistic	*p*
Normalized gray matter volume, L	0.73 ± 0.04	0.77 ± 0.04	12.05^c^	.001
Normalized white matter volume, L	0.67 ± 0.04	0.70 ± 0.04	15.04^c^	<.001
Hippocampus left, ml^a^	5.02 (4.37–5.33)	5.47 (5.11–5.78)	287.00^d^	.001
Hippocampus right, ml^a^	5.03 (4.33–5.45)	5.51 (5.10–5.86)	340.00^d^	.008
Number of hippocampal lesions^a^	1.00 (0.00–2.00)^b^	–	–	–
White matter lesion volume, ml^a^	4.22 (2.48–7.76)	–	–	–

Displayed data are mean ± *SD* for normally distributed variables. For non‐normally distributed data, median (interquartile range) is provided.

a Non‐normally distributed variable.

b *n* = 37.

c *F*‐value.

d *U*‐value.

### 3.3 Functional MRI group differences

The MS group performed worse on the fMRI paradigm compared to HCs (median MS patients: 35.00, interquartile range: 24.75–39.00; median HCs: 40.00, interquartile range: 35.00–42.50; *p *=* *.004; see Table 2). No significant difference was found for hippocampal activation during encoding of correctly remembered items between both groups (*p *>* *.05; see Table 3). Patients with MS had on average an equal amount of atlas regions as HCs (*p *=* *.076). Furthermore, patients did not show significant differences in sFC and dFC of the entire brain or the hippocampus compared to HCs (*p *>* *.05; see Table 3).

**Table 3:** Functional MRI results for patients with multiple sclerosis and healthy controls

	MS patients (*n *=* *38)	HCs (*n *=* *29)	Test statistic	*p*
Average motion, mm	0.10 ± 0.04	0.09 ± 0.04	1.14^d^	.290
Correctly remembered landscape images^a^	35.00 (24.75–39.00)	40.00 (35.00–42.50)	324.00^e^	.004
Number of atlas regions^a^	92.00 (90.00–92.00)	92.00 (91.50–92.00)	431.00^e^	.076
Task‐related activation (Z‐value)				
Hippocampus left	0.51 ± 0.70	0.52 ± 0.63	0.01^d^	.942
Hippocampus right	0.41 ± 0.72	0.47 ± 0.84	0.12^d^	.733
sFC				
Whole brain	0.32 ± 0.06	0.33 ± 0.08	0.32^d^	.573
Hippocampus left^b^	0.90 ± 0.23	0.87 ± 0.22	0.29^d^	.593
Hippocampus right^b^	0.90 ± 0.22	0.83 ± 0.19	1.84^d^	.180
dFC				
Whole brain	3.16 ± 0.25	3.18 ± 0.27	0.003^d^	.954
Hippocampus left^c^	0.98 ± 0.05	0.97 ± 0.05	0.28^d^	.599
Hippocampus right^c^	0.96 ± 0.05	0.98 ± 0.07	1.48^d^	.228

Displayed data are mean ± *SD* for normally distributed variables. For non‐normally distributed data, median (interquartile range) is provided.

dFC, dynamic functional connectivity; sFC, stationary functional connectivity.

a Not normally distributed variable.

b Values are corrected for within‐subject whole‐brain sFC.

c Values are corrected for within‐subject whole‐brain dFC.

d *F*‐value.

e *U*‐value.

In patients, correlation analyses revealed that hippocampal dFC was not related to hippocampal sFC or activation (*p > *.413), except for dFC and sFC of the left hippocampus (*r* = .39, *p *=* *.017). For HCs, only dFC of the right hippocampus was related to sFC of the left hippocampus (*r *=* *−.38, *p *=* *.041), whereas all other relationships were not significant (*p* > .433). Furthermore, in MS dFC was not significantly correlated with hippocampal volume, WM lesion volume, head motion, disease duration, anxiety, depression, or fatigue (*p *≥* *.208).

### 3.4 Predicting learning and memory performance in patients with MS

First, regression analyses were run in patients with MS including confounding variables (block 1) and traditional hippocampal measures (block 2–4) only, in order to predict verbal and visuospatial learning and memory performance separately (see Table 4). Next, dFC (block 5) was entered into the regression analysis to quantify the addition in explained variance in learning and memory performance (see Table 4).

**Table 4:** Significant predictors of learning and memory performance in patients with multiple sclerosis

Predictor	Adjusted *R* ^2^	Standardized β	Test statistic	*p*
Verbal learning and memory				
*block 1–4 total model*	.46	–	16.65^a^	<.001
Female sex	–	0.63	5.23^b^	<.001
Volume hippocampus left	–	0.29	2.38^b^	.023
*block 1–5 total model*	.53	–	14.61^a^	<.001
Female sex	–	0.54	4.45^b^	<.001
Volume hippocampus left	–	0.37	3.10^b^	.004
dFC hippocampus left	–	−0.30	−2.42^b^	.021
Visuospatial learning and memory				
*block 1–4 total model*	.11	–	5.70^a^	.022
sFC hippocampus right	–	0.37	2.39^b^	.022
*block 1–5 total model*	.24	–	6.72^a^	.003
sFC hippocampus right	–	0.32	2.19^b^	.035
dFC hippocampus right	–	−0.38	−2.61^b^	.013

dFC, dynamic functional connectivity; sFC, stationary functional connectivity.

a *F*‐value.

b *t*‐value.

#### 3.4.1 Verbal learning and memory

**Figure 2:**
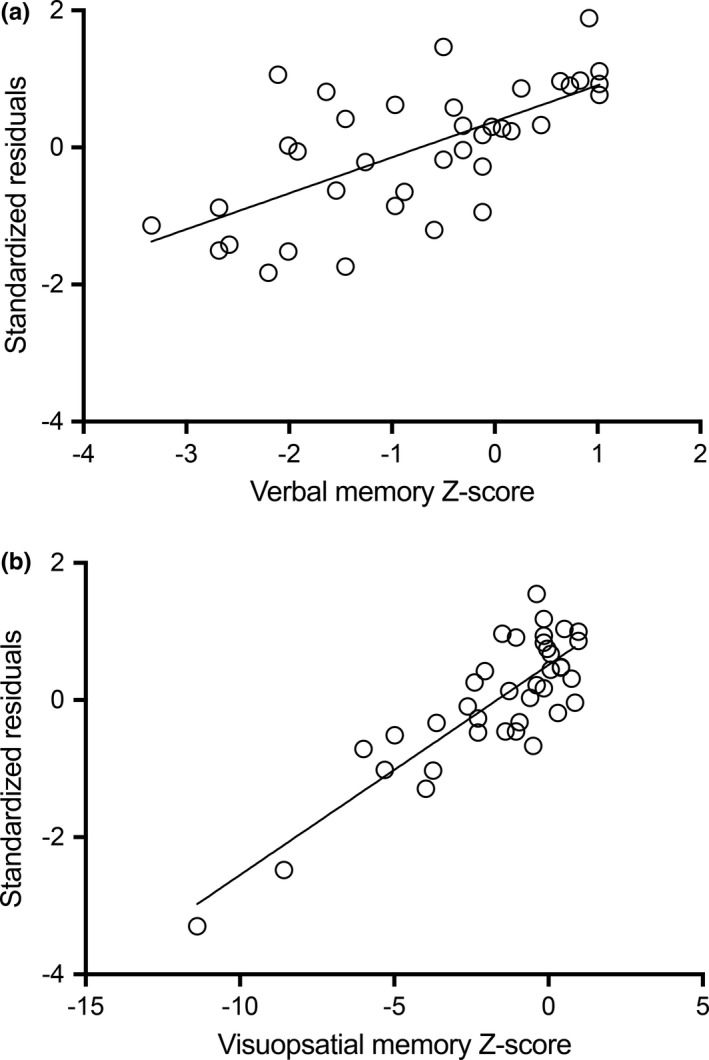
Standardized residuals of regression models. For both verbal learning and memory (a) and visuospatial learning and memory (b), the standardized residuals of the regression model including dynamic functional connectivity (dFC) measures are plotted against the Z‐scores of each memory test

For verbal learning and memory, lower left hippocampal dFC was correlated with better performance outside the scanner. Adding dFC of the hippocampus to the regression analysis led to a 7% increase in explained variance (*F *=* *5.88; *p *=* *.021). In total, 53% of variance (*F *=* *14.61; *p *<* *.001) could be explained by female sex (standardized β = 0.54; *p *<* *.001), left NHV (standardized β = 0.37; *p *<* *.001), and left hippocampal dFC (standardized β = −0.30; *p *=* *.021; see Figure 2a for the standardized residuals plot). In HCs, 43% of variance in verbal learning and memory could be explained by female sex (standardized β = 0.33; *p *=* *.034) and educational level (standardized β = 0.53; *p *=* *.001).

#### 3.4.2 Visuospatial learning and memory

**Figure 3:**
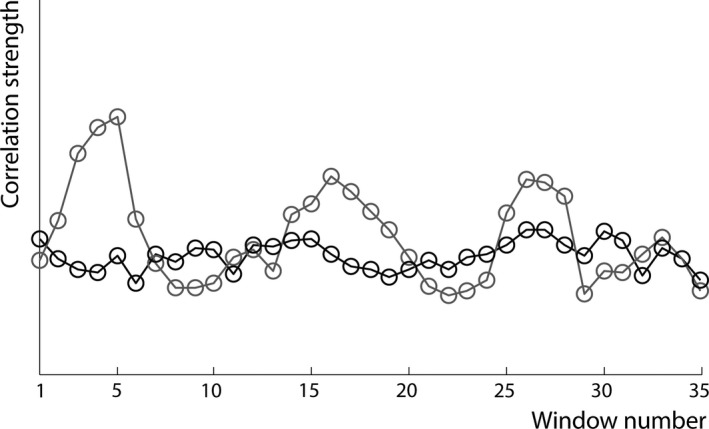
High and low dynamic functional connectivity (dFC) of two patients with multiple sclerosis. For illustrative purposes, the average stationary functional connectivity (*y*‐axis) of the left hippocampus with the rest of the brain is plotted for each consecutive window (*x*‐axis). The gray line represents a multiple sclerosis patient with high dFC during the memory task, while the black line represents a patient with low dFC

For visuospatial learning and memory, a similar association was found, but now for dFC of the right hippocampus. An extra 13% of variance could be explained by adding hippocampal dFC to the regression analysis (*F *=* *6.82; *p *=* *.013). In total, 24% of variance in visuospatial learning and memory performance (*F *=* *6.72; *p *=* *.003) could be explained by sFC of the right hippocampus (standardized β = 0.37; *p *=* *.035), and dFC of the right hippocampus (standardized β = −0.38; *p *=* *.013; see Figure 2b for the standardized residuals plot). For illustrative purposes, Figure 3 and Video S1 display one patient with low dFC of the left hippocampus and one patient with high dFC. In HCs, 44% of variance could be explained by age (standardized β = 0.33; *p *=* *.033), depression score (standardized β = 0.66; *p *<* *.001), and sFC of the right hippocampus (standardized β = −0.44; *p *=* *.013).

**Figure 4:**
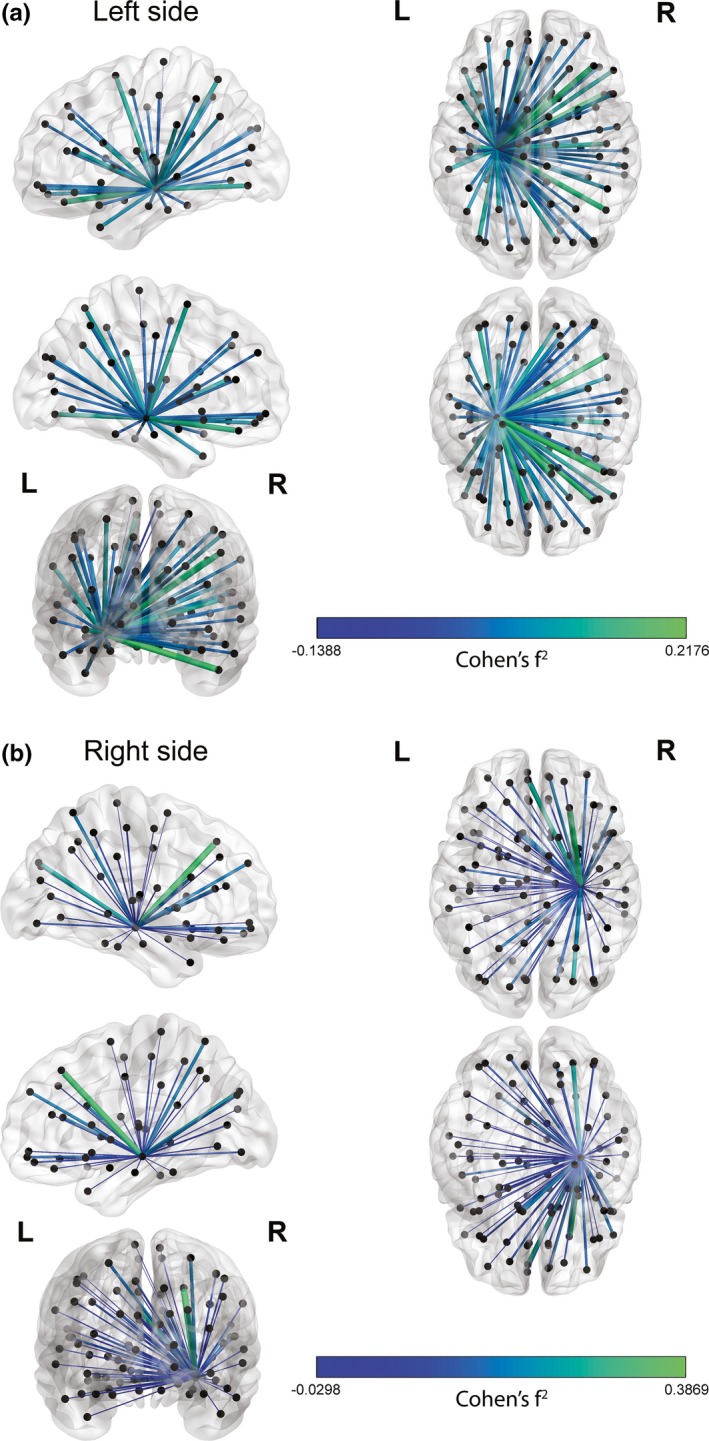
Visualization of spatial importance of dynamic functional connectivity (dFC) for learning and memory in multiple sclerosis. For verbal (a) and visuospatial (b) memory separately, the increase in effect size as a result of adding dFC on top of stationary brain measures (sex and left hippocampal volume for verbal learning and memory, and stationary functional connectivity of the right hippocampus for visuospatial learning and memory) is projected on a glass brain using BrainNet Viewer. A positive value suggests that dFC increases the effect size on top of stationary brain measures, whereas a negative value indicates a decrease in effect size

Figure 4 visualizes the results from the post hoc analyses, were we explored the spatial importance of dFC of the left and right hippocampus for verbal and visuospatial learning and memory in MS. Upon visual inspection, dynamic connections between the hippocampus and visual areas, as well as frontal regions, seem to be important for memory performance (note that these analyses were not statistically tested, but merely used for displaying purposes).

#### 3.4.3 Specificity analyses

In MS, thalamic dFC was not a significant predictor for verbal and visuospatial learning and memory (in isolation or next to dFC of the hippocampus). Additionally, NGMV was only a significant predictor for visuospatial memory (standardized β = 0.302, *p *=* *.036) besides sFC and dFC of the right hippocampus. NWMV was not a significant predictor (see Table S1). Finally, 35% of variance in average cognitive functioning could be explained by female sex (standardized β = 0.435, *p *=* *.004) and left hippocampal volume (standardized β = 0.425, *p *=* *.004; see Table S2).

## 4 DISCUSSION

In line with our hypothesis, we found that task‐related dFC is an important additional correlate of learning and memory function in patients with MS. Dynamic FC of the hippocampus explained an additional 7 and 13 percent of variance in verbal and visuospatial learning and memory performance outside the scanner, respectively, even on top of traditional measures of NHV, activation, and sFC. For both verbal and visuospatial learning and memory, lower dFC of the hippocampus specifically was related to better performance. For verbal learning and memory, dFC of the left hippocampus and its volume remained in the model as significant predictors, whereas other stationary functional measures did not, indicating that relatively low dFC in combination with little atrophy reflects a beneficial cognitive phenotype in MS. For visuospatial learning and memory, sFC of the right hippocampus remained in the model as a significant predictor, together with dFC of the right hippocampus. This suggests that strong (high sFC) and stable (low dFC) right‐lateralized hippocampal connectivity with other brain regions is beneficial for visuospatial learning and memory function in patients with MS.

Although no differences were found in dFC and sFC at the group level, in HCs a negative correlation was observed between dFC of the right hippocampus and sFC of the left hippocampus, which was not found in MS. In contrast, in the MS group, a positive relationship was observed between dFC and sFC of the left hippocampus, suggesting that MS might alter the interplay between different functional brain measures. Furthermore, in MS dFC was not significantly correlated with hippocampal volume, WM lesion volume, head motion, disease duration, anxiety, depression or fatigue. This suggests that the relationships we observed between hippocampal dFC with memory function are not simply a reflection of disease progression or related to symptoms of depression or fatigue. Irrespective of the complex relationship between dFC and sFC in MS, dFC did explain unique variance in learning and memory.

The relationship between brain dynamics and cognitive function is complex and likely depends on both brain state and cognitive domain. With respect to state, our findings are in line with a previous study showing low levels of dFC, but high levels of sFC, during task engagement, whereas at rest the brain displayed an opposite pattern (Hellyer et al., 2016). Furthermore, it could be hypothesized that certain cognitive domains benefit more from high levels of dFC than others. For example, execution of a complex task (i.e., cognitive flexibility) and information processing speed intuitively rely on the ability of the brain to rapidly change its connectivity pattern to optimize information transfer. This has previously been shown by several studies that indeed linked higher levels of brain dynamics to better executive functioning or information processing speed in healthy subjects (Braun et al., 2015; Cole et al., 2013; Douw et al., 2016; Nomi et al., 2016). The present results suggest this does not hold true for learning and memory in MS patients. Previous studies on brain dynamics and motor learning, recognition, and attention, hypothesize that the brain consists of a “rigid core” and “flexible periphery.”(Bassett et al., 2011, 2013; Telesford et al., 2016) This core‐periphery distinction was made by identifying how particular brain regions alter their modular allegiance, that is, community structure, over time. Low‐dynamic nodes (i.e., rigid core) mainly consisted of sensorimotor and visual regions, whereas high‐dynamic nodes (i.e., flexible periphery) mostly contained multimodal association areas (Bassett et al., 2011, 2013). The remaining nodes (i.e., the “bulk”) mostly included frontal and temporal regions, including the hippocampus (Bassett et al., 2013). Subjects with a more rigid core and more flexible periphery were better able to learn a motor task than subjects with a less rigid core and flexible periphery (Bassett et al., 2013). Recent insights suggest that the “bulk” becomes more important for controlling different brain states linked to cognitive processes when the brain is damaged (i.e., a possible compensatory role) (Betzel, Gu, Medaglia, Pasqualetti, & Bassett, 2016). From this perspective, it could be speculated that our results reflect an exaggeration of the contrast between core and periphery dynamics in MS, such that MS patients with preserved memory functioning already had or have developed lower hippocampal dynamics than those with impaired memory. This hypothesis is all the more interesting, as we did not observe any group‐level differences in dFC between patients and controls.

An alternative explanation for our results could be that lower hippocampal dFC is beneficial during encoding, by maintaining more stable connections with remote brain regions. However, in HCs we found that for visuospatial memory lower sFC of the right hippocampus was a significant predictor, whereas in MS an opposite relationship was found, suggesting that the interplay between functional measures and behavior might be altered in MS. Interestingly, a previous study that observed altered dynamics of parietal and prefrontal regions at rest in minimally disabled relapsing‐remitting MS patients did not show a relationship with a clinical outcome measure (Expanded Disability Status Scale), possibly explained by limited variation in physical disability due to selection bias (Leonardi et al., 2013). Important to take into account are large differences in operationalization of dynamics and task paradigms across studies, making comparisons between studies challenging.

The observation that associations between learning and memory performance and hippocampal dFC were content‐specific in terms of lateralization further supports our idea that dynamics are necessary to better understand cognitive (dys)functioning in MS patients. Furthermore, we showed that dFC of the thalamus, a structure that is often affected in MS, was not a significant predictor for memory function (Kipp et al., 2015). NGMV did remain in the model next to hippocampal measures when predicting visuospatial memory, while NWMV was not a significant predictor for memory function. Furthermore, dFC of the hippocampus was not a significant predictor for average cognitive functioning. Together, these analyses highlight the specificity of the present results linking dynamic hippocampal measures to memory performance and also show that the relationship between dFC and cognition is spatially modulated by the specific cognitive domain (visual vs verbal learning and memory). For visuospatial learning and memory, dFC of the right hippocampus remained in the model together with sFC of the right hippocampus. That sFC remained in the model might be explained by the fMRI paradigm we used, which was more visuospatially than verbally oriented, and may have rendered the relationship between functional measures and visuospatial memory outside the scanner conceptually stronger to that with verbal memory. For verbal learning and memory, left hippocampal volume remained in the prediction model besides dFC of the left hippocampus. Both dFC and sFC of the left hippocampus were moderately correlated with each other in the MS group. However, only dFC was included in the final model predicting verbal memory, which suggests that it explains more variance than sFC. Although lateralization was observed, the spatial exploration of the relative importance of dFC with respect to learning and memory on top of stationary measures revealed that dFC between the hippocampus and especially visual and frontal cortices were important for both verbal and visuospatial memory.

Unfortunately, we were not able to investigate dynamics of the hippocampus with the entire brain, as image acquisition was optimized toward the hippocampus (i.e., the part superior to the cingulate cortex was not included). Although no significant group differences were found in terms of the number and location of brain regions that were excluded in the sFC and dFC analysis, our study would have been more complete if we could have included superior cortical regions as well. Nevertheless, we do not expect a large effect of partial brain coverage on our present results, as we averaged dFC of the hippocampus over all its connections for each subject and were thus relatively insensitive to spatial effects. Second, we did not perform lesion filling, as our main focus was on functional brain measures. Hence, the volumetric measures might not be as accurate compared to lesion‐filled measures. Third, eight patients had secondary progressive MS, whereas all others had relapsing‐remitting MS. Unfortunately, this small size of the secondary progressive MS group did not allow us to investigate possible effects of disease phase on hippocampal and behavioral measures. However, one can imagine that a patient's disease course might affect hippocampal dFC and memory performance, and perhaps mediate the link between both. Fourth, we decided not to include the number of hippocampal lesions in the regression analyses because of the following reasons: (1) one patient did not have a DIR image, which would further reduce our already small sample size (not favorable from a statistical perspective); (2) the number of predictors would increase, which is, again, not favorable in combination with a decrease in sample size, and; (3) hippocampal atrophy can be more accurately measured than hippocampal lesion load (i.e., hippocampal lesions are difficult to measure in volume on 1.5T and were therefore counted). Finally, the present, explorative, study may suffer from multiple testing problems when predicting learning and memory performance. By only including predictors relevant for the dependent variable and using a stepwise approach with forward selection, we believe we minimized the influence of multiple testing on the validity of our results.

Future studies should investigate the effect of disease course on dFC and its link with cognitive functioning in a larger sample, but also the exact interplay between conventional brain measures (including hippocampal lesions) and dynamic brain measures. Furthermore, other cognitive paradigms should be investigated to see whether the present findings are specific for the fMRI task we used and the cognitive domain that was tested outside the scanner. It would also be interesting to investigate dynamics and cognition across different neurological diseases, to see whether patterns of increased and decreased dynamics in relation to cognitive functioning are universal or disease‐specific.

To conclude, brain dynamics have not yet been explored extensively in MS, but seem to be an important feature of the biological mechanisms underlying learning and memory (dys)function. That is, lower task‐related dFC of the hippocampus, in combination with larger volume or higher sFC, is related to better verbal and visuospatial learning and memory performance outside the MR scanner in MS. This study highlights the relevance of brain dynamics on top of other, more traditional, MRI measures to understand cognitive (dys)function in MS.

## DISCLOSURES

van Geest has nothing to disclose. Hulst receives research support from the Dutch MS Research Foundation, grant number 08‐648 and serves as a consultant for Genzyme, Merck‐Serono, Teva Pharmaceuticals, and Novartis. Ms. Meijer reports no disclosures and receives research support from a research grant from Biogen Idec. Ms. Hoyng has nothing to disclose. Geurts serves on the editorial boards of MS Journal, BMC Neurology, MS International and Neurology and the Scientific Advisory Board of the Dutch MS Research Foundation and of MS Academia, Merck‐Serono, and has served as a consultant for Merck‐Serono, Biogen Idec, Novartis, Genzyme, and Teva Pharmaceuticals. Douw receives research support from a Branco Weiss Fellowship from Society in Science.

## References

[brb3954-bib-0001] Avila, C. , Barros‐Loscertales, A. , Forn, C. , Mallo, R. , Parcet, M. A. , Belloch, V. , … González‐Darder, J. M. (2006). Memory lateralization with 2 functional MR imaging tasks in patients with lesions in the temporal lobe. AJNR. American Journal of Neuroradiology, 27, 498–503. 16551984 PMC7976998

[brb3954-bib-0002] Bassett, D. S. , Wymbs, N. F. , Porter, M. A. , Mucha, P. J. , Carlson, J. M. , & Grafton, S. T. (2011). Dynamic reconfiguration of human brain networks during learning. Proceedings of the National Academy of Sciences of the United States of America, 108, 7641–7646. https://doi.org/10.1073/pnas.1018985108 21502525 10.1073/pnas.1018985108 PMC3088578

[brb3954-bib-0003] Bassett, D. S. , Wymbs, N. F. , Rombach, M. P. , Porter, M. A. , Mucha, P. J. , & Grafton, S. T. (2013). Task‐based core‐periphery organization of human brain dynamics. PLoS Computational Biology, 9, e1003171 https://doi.org/10.1371/journal.pcbi.1003171 24086116 10.1371/journal.pcbi.1003171 PMC3784512

[brb3954-bib-0004] Beckmann, C. F. , Jenkinson, M. , & Smith, S. M. (2003). General multilevel linear modeling for group analysis in FMRI. NeuroImage, 20, 1052–1063. https://doi.org/10.1016/S1053-8119(03)00435-X 14568475 10.1016/S1053-8119(03)00435-X

[brb3954-bib-0005] Beckmann, C. F. , & Smith, S. M. (2004). Probabilistic independent component analysis for functional magnetic resonance imaging. IEEE Transactions on Medical Imaging, 23, 137–152. https://doi.org/10.1109/TMI.2003.822821 14964560 10.1109/TMI.2003.822821

[brb3954-bib-0006] Benedict, R. H. , Ramasamy, D. , Munschauer, F. , Weinstock‐Guttman, B. , & Zivadinov, R. (2009). Memory impairment in multiple sclerosis: Correlation with deep grey matter and mesial temporal atrophy. Journal of Neurology, Neurosurgery and Psychiatry, 80, 201–206. https://doi.org/10.1136/jnnp.2008.148403 10.1136/jnnp.2008.148403 18829629

[brb3954-bib-0007] Betzel, R. F. , Gu, S. , Medaglia, J. D. , Pasqualetti, F. , & Bassett, D. S. (2016). Optimally controlling the human connectome: The role of network topology. Scientific Reports, 6, 30770 https://doi.org/10.1038/srep30770 27468904 10.1038/srep30770 PMC4965758

[brb3954-bib-0008] Braun, U. , Schafer, A. , Walter, H. , Erk, S. , Romanczuk‐Seiferth, N. , Haddad, L. , … Bassett, D. S. (2015). Dynamic reconfiguration of frontal brain networks during executive cognition in humans. Proceedings of the National Academy of Sciences of the United States of America, 112, 11678–11683. https://doi.org/10.1073/pnas.1422487112 26324898 10.1073/pnas.1422487112 PMC4577153

[brb3954-bib-0009] Bucks, R. S. , & Willison, J. R. (1997). Development and validation of the location learning test (LLT): A test of visuo‐spatial learning designed for use with older adults and in dementia. The Clinical Neuropsychologist, 11, 273–286. https://doi.org/10.1080/13854049708400456

[brb3954-bib-0010] Chen, X. , Zhang, H. , Zhang, L. , Shen, C. , Lee, S. W. , & Shen, D. (2017). Extraction of dynamic functional connectivity from brain grey matter and white matter for MCI classification. Human Brain Mapping, 38(10), 5019–5034. https://doi.org/10.1002/hbm.23711 28665045 10.1002/hbm.23711 PMC5593789

[brb3954-bib-0011] Chiaravalloti, N. D. , & DeLuca, J. (2008). Cognitive impairment in multiple sclerosis. The Lancet Neurology, 7, 1139–1151. https://doi.org/10.1016/S1474-4422(08)70259-X 19007738 10.1016/S1474-4422(08)70259-X

[brb3954-bib-0012] Cole, M. W. , Reynolds, J. R. , Power, J. D. , Repovs, G. , Anticevic, A. , & Braver, T. S. (2013). Multi‐task connectivity reveals flexible hubs for adaptive task control. Nature Neuroscience, 16, 1348–1355. https://doi.org/10.1038/nn.3470 23892552 10.1038/nn.3470 PMC3758404

[brb3954-bib-0013] Daselaar, S. M. , Veltman, D. J. , Rombouts, S. A. , Raaijmakers, J. G. , & Jonker, C. (2003). Deep processing activates the medial temporal lobe in young but not in old adults. Neurobiology of Aging, 24, 1005–1011. https://doi.org/10.1016/S0197-4580(03)00032-0 12928060 10.1016/s0197-4580(03)00032-0

[brb3954-bib-0014] Douw, L. , Leveroni, C. L. , Tanaka, N. , Emerton, B. C. , Cole, A. C. , Reinsberger, C. , Stufflebeam, S. M. (2015). Loss of resting‐state posterior cingulate flexibility is associated with memory disturbance in left temporal lobe epilepsy. PLoS ONE, 10, e0131209 https://doi.org/10.1371/journal.pone.0131209 26110431 10.1371/journal.pone.0131209 PMC4481466

[brb3954-bib-0015] Douw, L. , Wakeman, D. G. , Tanaka, N. , Liu, H. , & Stufflebeam, S. M. (2016). State‐dependent variability of dynamic functional connectivity between frontoparietal and default networks relates to cognitive flexibility. Neuroscience, 339, 12–21. https://doi.org/10.1016/j.neuroscience.2016.09.034 27687802 10.1016/j.neuroscience.2016.09.034 PMC5635855

[brb3954-bib-0016] Hellyer, P. J. , Jachs, B. , Clopath, C. , & Leech, R. (2016). Local inhibitory plasticity tunes macroscopic brain dynamics and allows the emergence of functional brain networks. NeuroImage, 124, 85–95. https://doi.org/10.1016/j.neuroimage.2015.08.069 26348562 10.1016/j.neuroimage.2015.08.069 PMC6684371

[brb3954-bib-0017] Hulst, H. E. , Schoonheim, M. M. , Roosendaal, S. D. , Popescu, V. , Schweren, L. J. , van der Werf, L. J. , … Geurts, J. J. (2012). Functional adaptive changes within the hippocampal memory system of patients with multiple sclerosis. Human Brain Mapping, 33, 2268–2280. https://doi.org/10.1002/hbm.21359 21898674 10.1002/hbm.21359 PMC6869948

[brb3954-bib-0018] Hulst, H. E. , Schoonheim, M. M. , Van, G. Q. , Uitdehaag, B. M. , Barkhof, F. , & Geurts, J. J. (2015). Memory impairment in multiple sclerosis: Relevance of hippocampal activation and hippocampal connectivity. Multiple Sclerosis, 21(13), 1705–1712. https://doi.org/10.1177/1352458514567727 25680986 10.1177/1352458514567727

[brb3954-bib-0019] Hutchison, R. M. , Womelsdorf, T. , Allen, E. A. , Bandettini, P. A. , Calhoun, V. D. , Corbetta, M. , … Chang, C. (2013). Dynamic functional connectivity: Promise, issues, and interpretations. NeuroImage, 80, 360–378. https://doi.org/10.1016/j.neuroimage.2013.05.079 23707587 10.1016/j.neuroimage.2013.05.079 PMC3807588

[brb3954-bib-0020] Igloi, K. , Doeller, C. F. , Berthoz, A. , Rondi‐Reig, L. , & Burgess, N. (2010). Lateralized human hippocampal activity predicts navigation based on sequence or place memory. Proceedings of the National Academy of Sciences of the United States of America, 107, 14466–14471. https://doi.org/10.1073/pnas.1004243107 20660746 10.1073/pnas.1004243107 PMC2922562

[brb3954-bib-0021] Jia, H. , Hu, X. , & Deshpande, G. (2014). Behavioral relevance of the dynamics of the functional brain connectome. Brain Connectivity, 4, 741–759. https://doi.org/10.1089/brain.2014.0300 25163490 10.1089/brain.2014.0300 PMC4238311

[brb3954-bib-0022] Kipp, M. , Wagenknecht, N. , Beyer, C. , Samer, S. , Wuerfel, J. , & Nikoubashman, O. (2015). Thalamus pathology in multiple sclerosis: From biology to clinical application. Cellular and Molecular Life Sciences, 72, 1127–1147. https://doi.org/10.1007/s00018-014-1787-9 25417212 10.1007/s00018-014-1787-9 PMC11113280

[brb3954-bib-0023] Lechner‐Scott, J. , Kappos, L. , Hofman, M. , Polman, C. H. , Ronner, H. , Montalban, X. , … Gibberd, R. (2003). Can the expanded disability status scale be assessed by telephone? Multiple Sclerosis, 9, 154–159. https://doi.org/10.1191/1352458503ms884oa 12708811 10.1191/1352458503ms884oa

[brb3954-bib-0024] Leonardi, N. , Richiardi, J. , Gschwind, M. , Simioni, S. , Annoni, J. M. , Schluep, M. , … Van De Ville, D. (2013). Principal components of functional connectivity: A new approach to study dynamic brain connectivity during rest. NeuroImage, 83, 937–950. https://doi.org/10.1016/j.neuroimage.2013.07.019 23872496 10.1016/j.neuroimage.2013.07.019

[brb3954-bib-0025] Leonardi, N. , Shirer, W. R. , Greicius, M. D. , & Van De Ville, D. (2014). Disentangling dynamic networks: Separated and joint expressions of functional connectivity patterns in time. Human Brain Mapping, 35, 5984–5995. https://doi.org/10.1002/hbm.22599 25081921 10.1002/hbm.22599 PMC6868958

[brb3954-bib-0026] Leonardi, N. , & Van De Ville, D. (2015). On spurious and real fluctuations of dynamic functional connectivity during rest. NeuroImage, 104, 430–436. https://doi.org/10.1016/j.neuroimage.2014.09.007 25234118 10.1016/j.neuroimage.2014.09.007

[brb3954-bib-0027] Mulder, J. L. , Dekker, P. H. , & Dekker, R. (1996). Verbale Leer‐ en Geheugentest. Lisse, The Netherlands: Swets and Zeitlinger.

[brb3954-bib-0028] Nguyen, T. T. , Kovacevic, S. , Dev, S. I. , Lu, K. , Liu, T. T. , & Eyler, L. T. (2016). Dynamic functional connectivity in bipolar disorder is associated with executive function and processing speed: A Preliminary Study. Neuropsychology, 31(1), 73–83. 27775400 10.1037/neu0000317 PMC5471616

[brb3954-bib-0029] Nomi, J. S. , Vij, S. G. , Dajani, D. R. , Steimke, R. , Damaraju, E. , Rachakonda, S. , … Uddin, L. Q. (2016). Chronnectomic patterns and neural flexibility underlie executive function. NeuroImage, 147, 861–871. 27777174 10.1016/j.neuroimage.2016.10.026 PMC5303676

[brb3954-bib-0030] Patenaude, B. , Smith, S. M. , Kennedy, D. N. , & Jenkinson, M. (2011). A Bayesian model of shape and appearance for subcortical brain segmentation. NeuroImage, 56, 907–922. https://doi.org/10.1016/j.neuroimage.2011.02.046 21352927 10.1016/j.neuroimage.2011.02.046 PMC3417233

[brb3954-bib-0031] Polman, C. H. , Reingold, S. C. , Banwell, B. , Clanet, M. , Cohen, J. A. , Filippi, M. , … Wolinsky, J. S. (2011). Diagnostic criteria for multiple sclerosis: 2010 revisions to the McDonald criteria. Annals of Neurology, 69, 292–302. https://doi.org/10.1002/ana.22366 21387374 10.1002/ana.22366 PMC3084507

[brb3954-bib-0032] Rao, S. M. , Leo, G. J. , Bernardin, L. , & Unverzagt, F. (1991). Cognitive dysfunction in multiple sclerosis. I. Frequency, patterns, and prediction. Neurology, 41, 685–691. https://doi.org/10.1212/WNL.41.5.685 2027484 10.1212/wnl.41.5.685

[brb3954-bib-0033] Mitchell, A. J. , Benito‐Leon, J. , Gonzalez, J. M. , & Rivera‐Navarro, J. (2005). Quality of life and its assessment in multiple sclerosis: Integrating physical and psychological components of wellbeing. The Lancet Neurology, 4, 556–566. https://doi.org/10.1016/S1474-4422(05)70166-6 16109362 10.1016/S1474-4422(05)70166-6

[brb3954-bib-0034] Roosendaal, S. D. , Hulst, H. E. , Vrenken, H. , Feenstra, H. E. , Castelijns, J. A. , Pouwels, P. J. , … Geurts, J. J. (2010). Structural and functional hippocampal changes in multiple sclerosis patients with intact memory function. Radiology, 255, 595–604. https://doi.org/10.1148/radiol.10091433 20413769 10.1148/radiol.10091433

[brb3954-bib-0035] Roosendaal, S. D. , Moraal, B. , Vrenken, H. , Castelijns, J. A. , Pouwels, P. J. , Barkhof, F. , Geurts, J. J. (2008). In vivo MR imaging of hippocampal lesions in multiple sclerosis. Journal of Magnetic Resonance Imaging, 27, 726–731. https://doi.org/10.1002/(ISSN)1522-2586 18302199 10.1002/jmri.21294

[brb3954-bib-0036] Smith, S. M. , Zhang, Y. , Jenkinson, M. , Chen, J. , Matthews, P. M. , Federico, A. , De Stefano, N. (2002). Accurate, robust, and automated longitudinal and cross‐sectional brain change analysis. NeuroImage, 17, 479–489. https://doi.org/10.1006/nimg.2002.1040 12482100 10.1006/nimg.2002.1040

[brb3954-bib-0037] Steenwijk, M. D. , Geurts, J. J. , Daams, M. , Tijms, B. M. , Wink, A. M. , Balk, L. J. , … Pouwels, P. J. (2016). Cortical atrophy patterns in multiple sclerosis are non‐random and clinically relevant. Brain, 139, 115–126. https://doi.org/10.1093/brain/awv337 26637488 10.1093/brain/awv337

[brb3954-bib-0038] Telesford, Q. K. , Lynall, M. E. , Vettel, J. , Miller, M. B. , Grafton, S. T. , & Bassett, D. S. (2016). Detection of functional brain network reconfiguration during task‐driven cognitive states. NeuroImage, 142, 198–210. https://doi.org/10.1016/j.neuroimage.2016.05.078 27261162 10.1016/j.neuroimage.2016.05.078 PMC5133201

[brb3954-bib-0039] Tzourio‐Mazoyer, N. , Landeau, B. , Papathanassiou, D. , Crivello, F. , Etard, O. , Delcroix, N. , … Joliot, M. (2002). Automated anatomical labeling of activations in SPM using a macroscopic anatomical parcellation of the MNI MRI single‐subject brain. NeuroImage, 15, 273–289. https://doi.org/10.1006/nimg.2001.0978 11771995 10.1006/nimg.2001.0978

[brb3954-bib-0040] Van Der Werf, Y. D. , Altena, E. , Schoonheim, M. M. , Sanz‐Arigita, E. J. , Vis, J. C. , De Rijke, W. , Van Someren, E. J. (2009). Sleep benefits subsequent hippocampal functioning. Nature Neuroscience, 12, 122–123. https://doi.org/10.1038/nn.2253 19151712 10.1038/nn.2253

[brb3954-bib-0041] Vercoulen, J. H. , Swanink, C. M. , Fennis, J. F. , Galama, J. M. , van der Meer, J. W. , & Bleijenberg, G. (1994). Dimensional assessment of chronic fatigue syndrome. Journal of Psychosomatic Research, 38, 383–392. https://doi.org/10.1016/0022-3999(94)90099-X 7965927 10.1016/0022-3999(94)90099-x

[brb3954-bib-0042] Xia, M. , Wang, J. , & He, Y. (2013). BrainNet Viewer: A network visualization tool for human brain connectomics. PLoS ONE, 8, e68910 https://doi.org/10.1371/journal.pone.0068910 23861951 10.1371/journal.pone.0068910 PMC3701683

[brb3954-bib-0043] Zigmond, A. S. , & Snaith, R. P. (1983). The hospital anxiety and depression scale. Acta Psychiatrica Scand., 67, 361–370.https://doi.org/10.1111/j.1600-0447.1983.tb09716.x 10.1111/j.1600-0447.1983.tb09716.x 6880820

