# Supplementary material for: The importance of hippocampal dynamic connectivity in explaining memory function in multiple sclerosis
Source: Brain Behav. 2018 Mar 30;8(5):e00954. doi: 10.1002/brb3.954 (PMC5943730; doi:10.1002/brb3.954)
Supplement: Supplementary file 1 [file BRB3-8-e00954-s001.docx]

**
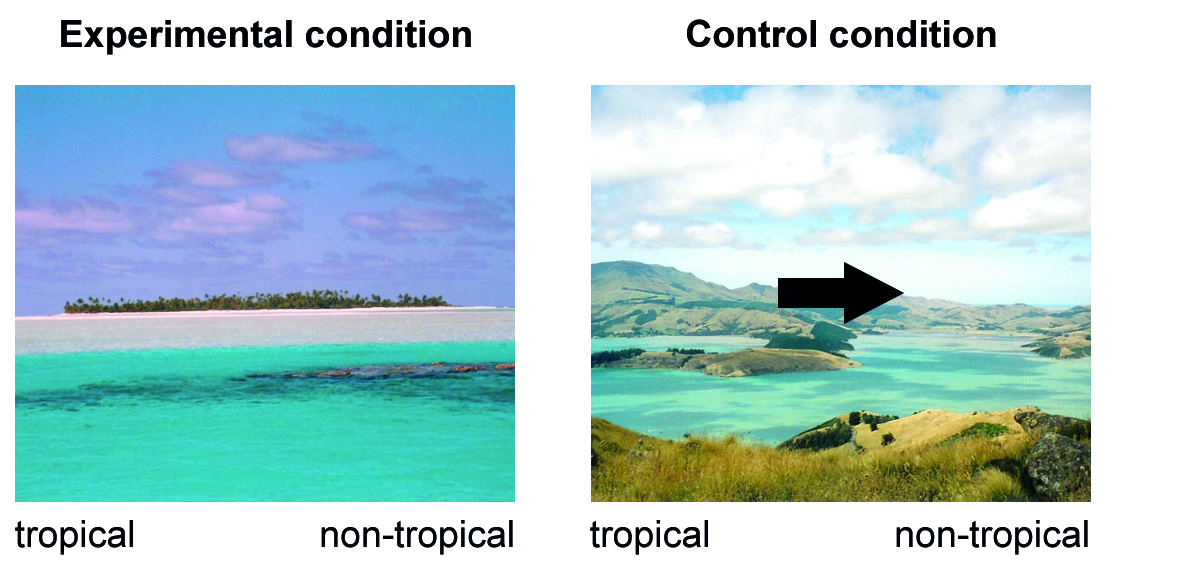
**

**Supplementary Figure 1. overview of fMRI paradigm**

In the experimental condition of the paradigm subjects had to indicate whether the landscape image is tropical or non-tropical. The experimental trials were alternated with control trials in which the subject had to indicate whether the arrow points to the left or right side. Note that the text beneath the images was identical in the experimental and control trials in order to rule out its possible effects on brain activation.
