# Supplementary material for: The importance of hippocampal dynamic connectivity in explaining memory function in multiple sclerosis
Source: Brain Behav. 2018 Mar 30;8(5):e00954. doi: 10.1002/brb3.954 (PMC5943730; doi:10.1002/brb3.954)
Supplement: Supplementary file 2 [file BRB3-8-e00954-s002.docx]

**Supplementary Table 2. Significant predictors for average cognitive functioning in patients with multiple sclerosis**

| **Predictor** | **Adjusted R^2^** | **Standardized β** | **Test statistic** | ***P*** |
| --- | --- | --- | --- | --- |
| *Z-score cognitive functioning* | | | | |
| *All block 1 – 5 total model* | 0.35 | – | 10.01^†^ | < 0.001 |
| Female sex | – | 0.44 | 3.13^††^ | 0.004 |
| Volume hippocampus left | – | 0.43 | 3.07^††^ | 0.004 |

^†^ *F*-value.

^††^ *t*-value.
