# Supplementary material for: The importance of hippocampal dynamic connectivity in explaining memory function in multiple sclerosis
Source: Brain Behav. 2018 Mar 30;8(5):e00954. doi: 10.1002/brb3.954 (PMC5943730; doi:10.1002/brb3.954)
Supplement: Supplementary file 3 [file BRB3-8-e00954-s003.docx]

**Supplementary Table 1. Significant predictors obtained from specificity analyses in patients with multiple sclerosis**

| **Predictor** | **Adjusted R^2^** | **Standardized β** | **Test statistic** | ***P*** |
| --- | --- | --- | --- | --- |
| *Verbal learning and memory* | | | | |
| *All blocks – dFC thalamus instead of dFC hippocampus* | 0.46 | – | 16.65^†^ | < 0.001 |
| Female sex | – | 0.63 | 5.23^††^ | < 0.001 |
| Volume hippocampus left | – | 0.29 | 2.38^††^ | 0.023 |
| *All blocks – dFC thalamus next to dFC hippocampus* | 0.53 | – | 14.61^†^ | < 0.001 |
| Female sex | – | 0.54 | 4.45^††^ | < 0.001 |
| Volume hippocampus left | – | 0.37 | 3.10^††^ | 0.004 |
| dFC hippocampus left | – | -0.30 | -2.42^††^ | 0.021 |
| *All blocks – NWMV next to dFC hippocampus* | 0.53 | – | 14.61^†^ | < 0.001 |
| Female sex | – | 0.54 | 4.45^††^ | < 0.001 |
| Volume hippocampus left | – | 0.37 | 3.10^††^ | 0.004 |
| dFC hippocampus left | – | -0.30 | -2.42^††^ | 0.021 |
| *All blocks – NGMV next to dFC hippocampus* | 0.53 | – | 14.61^†^ | < 0.001 |
| Female sex | – | 0.54 | 4.45^††^ | < 0.001 |
| Volume hippocampus left | – | 0.37 | 3.10^††^ | 0.004 |
| dFC hippocampus left | – | -0.30 | -2.42^††^ | 0.021 |
| *Visuospatial learning and memory* | | | | |
| *All blocks – dFC thalamus instead of dFC hippocampus* | 0.11 | – | 5.70^†^ | 0.022 |
| sFC hippocampus right | – | 0.37 | 2.39^††^ | 0.022 |
| *All blocks – dFC thalamus next to dFC hippocampus* | 0.24 | – | 6.72^†^ | 0.003 |
| sFC hippocampus right | – | 0.32 | 2.19^††^ | 0.035 |
| dFC hippocampus right | – | -0.38 | -2.61^††^ | 0.013 |
| *All blocks – NWMV next to dFC hippocampus* | 0.24 | – | 6.72^†^ | 0.003 |
| sFC hippocampus right | – | 0.32 | 2.19^††^ | 0.035 |
| dFC hippocampus right | – | -0.38 | -2.61^††^ | 0.013 |
| *All blocks – NGMV next to dFC hippocampus* | 0.31 | – | 6.55^†^ | 0.001 |
| sFC hippocampus right | – | 0.29 | 2.08^††^ | 0.046 |
| dFC hippocampus right | – | -0.34 | -2.44^††^ | 0.020 |
| NGMV | – | 0.30 | 2.18^††^ | 0.036 |

dFC = dynamic functional connectivity; NGMV = normalized gray matter volume; NWMV = normalized white matter volume; sFC = stationary functional connectivity.

^†^ *F*-value.

^††^ *t*-value.
